# ASSOCIATIONS BETWEEN INTERPRETER DEPENDENCY AND REHABILITATION OUTCOMES AND CARE PROCESSES FOLLOWING ACQUIRED BRAIN INJURY: A SWEDISH REGISTRY-BASED COHORT STUDY

**DOI:** 10.2340/jrm.v58.45146

**Published:** 2026-09-14

**Authors:** Ellen GRUT, Monica BLOM JOHANSSON, Monika LÖFGREN

**Affiliations:** 1 Department of Rehabilitation Medicine, Danderyd University Hospital, Stockholm, Sweden; 2 Department of Public Health and Caring Sciences, Uppsala University, Uppsala, Sweden; 3 Department of Clinical Sciences, Danderyd hospital, Karolinska Institutet, Stockholm, Sweden

**Keywords:** acquired brain injury, interpreter, cultural and linguistic diversity, communication barriers, rehabilitation outcomes, process measures

## Abstract

**Objective:**

To explore interpreter dependency in relation to rehabilitation outcomes and rehabilitation process measures for patients with acquired brain injury.

**Design:**

Retrospective cohort study.

**Subjects:**

Data from 13,900 inpatients, with acquired brain injury as main diagnosis, included in a Swedish quality registry (2008–2020). Of these, 391 patients were interpreter-dependent whereas 13,509 were not.

**Method:**

Outcomes were functional independence, health-related quality of life, self-perceived health, occurrence of complications, and length of stay. Process measures consisted of establishment of an individualized rehabilitation plan and collection of patient-reported experience measures.

**Results:**

On admission as well as discharge, interpreter dependency was related to lower scores regarding functional independence, health-related quality of life, and self-perceived health, while showing no association with the degree of improvement in these outcomes between the 2 time points. Interpreter-dependent patients were less likely to have had patient-reported experience measures administered. The results regarding functional independence and patient-reported experience measures persisted after adjusting for confounding variables. Length of stay, occurrence of complications, and establishment of an individualized rehabilitation plan were not related to interpreter dependency.

**Conclusion:**

Disparities on admission and discharge were evident regarding functionality as well as health-related quality of life and self-perceived health. It is possible that acquired brain injury severity or disparities earlier in the care continuum may underlie the results.

## LAY ABSTRACT

This study explored how the need for an interpreter affects rehabilitation outcomes for people with acquired brain injury in Sweden. The aim was to find out whether patients who require an interpreter during their care have different rehabilitation results compared with those who do not. Data were collected from 13,900 inpatients treated for acquired brain injury between 2008 and 2020 who had agreed to participate in a national quality registry. Among them, 391 patients were interpreter dependent, meaning they needed an interpreter to communicate with healthcare staff. On both admission and discharge, interpreter-dependent patients showed lower levels of functional independence, health-related quality of life, and self-perceived health compared with patients who did not need an interpreter. Both groups improved in function, quality of life, and self-perceived health during rehabilitation. The interpreter-dependent patients were less likely to have completed patient experience questionnaires. Interpreter dependency was not related to the length of hospital stay, the occurrence of complications, or the establishment of an individualized rehabilitation plan. Overall, the study found clear differences between interpreter-dependent and non-interpreter-dependent patients in terms of function and well-being on both admission and discharge. These differences might be due to the severity of the brain injury or to disparities earlier in the healthcare process.

As the foreign-born population in Sweden has increased over the last decade, there are increased demands to overcome language discordance within the healthcare system. In 2024, over 2 million people in Sweden were foreign born, i.e., 20 % of the population (1). According to the Health Care Act, Swedish healthcare must be provided on equal terms for the entire population (2). However, language barriers in healthcare have been shown to compromise both the quality and safety of care (3, 4). In Sweden, interpreter services are commonly used to overcome these barriers, but interpreter-mediated communication is not without challenges (5). Research highlights challenges such as unclear role expectations, complicated medical assessments (6), inconsistent access to and quality of interpreter services (7, 8), and variation in how interpreters are used across healthcare professions and settings (9, 10).

In the context of acquired brain injury (ABI), effective communication is essential for successful rehabilitation. ABI can lead to impairments of motor, sensory, and cognitive functions (11) and independence in daily life is a central goal in neurorehabilitation (12). Rehabilitation for ABI patients is often multidisciplinary, complex, and places high demands on communication (13). ABI patients with low proficiency in the majority language have been shown to experience disparities in healthcare regarding preventive treatment, length of stay (LOS), quality of care, and quality of life (14, 15). Similarly, interpreter need has been associated with lower independence after ABI, longer LOS, and reduced likelihood of receiving mood assessment (16, 17). Given the close interconnection between language, communication, and culture, it is essential to consider culture when studying linguistic diversity in healthcare. Internationally, cultural and linguistic diversity (CALD) has been associated with earlier onset of stroke and increased adverse outcomes (18) and a higher risk of sustaining ABI (19). These disparities are often compounded by low socioeconomic status, which is a risk factor for stroke, a determinant in access to specialized care (20) and long-term survival (21).

Despite reported challenges, numerous studies emphasize the importance of interpreter use in ensuring patient safety, improving medical outcomes, and enhancing quality of care (22–24). In ABI rehabilitation, interpreter use has been linked to greater rehabilitation gains, lower readmission rates, and higher quality of care (13, 25). Interpreter services have also been linked to more efficient healthcare use, potentially reducing long-term cost (26).

Along with the increase in interpreter-mediated care in Swedish healthcare, the ageing population is expected to have a significant impact on the number of patients affected by stroke (27), further increasing the demand for accessible, language-inclusive care. Yet, there is limited knowledge about how well interpreter-mediated neurorehabilitation aligns with the legal requirement for equitable care. Furthermore, a vast majority of published studies originate from the United States, where healthcare is largely insurance-based, in contrast to Sweden’s predominantly tax-funded system. In a globalized world where language diversity characterizes the clinical setting, linguistic accessibility and cultural understanding are central issues. There is also a need for a deeper understanding of rehabilitation outcomes for interpreter-mediated care in countries with diverse economic and demographic conditions.

## Aims

The aim of this study was to explore interpreter dependency in relation to rehabilitation outcomes and selected rehabilitation process measures for ABI patients participating in a national quality registry for rehabilitation medicine. Specifically, rehabilitation outcomes consisted of (*i*) Independence in daily life; (*ii*) Health-related quality of life (HRQL) and self-perceived health; (*iii*) Occurrence of complications, and (*iv*) Length of stay (LOS). The selected rehabilitation process measures consisted of (*v*) Establishment of individualized rehabilitation plan (IRP) and (*vi*) Collection of patient-reported experience measures (PREMs).

An additional aim was to explore degree of improvement from admission to discharge, regarding independence in daily life, HRQL, and self-perceived health.

## METHODS

### Design

This study is a registry-based retrospective cohort study.

### The Swedish registry for rehabilitation medicine (SveReh)

The Swedish registry for rehabilitation medicine (SveReh) is a national quality registry for neurological diseases and impairments (28). Clinics participating in the registry provide multidisciplinary rehabilitation in inpatient and/or outpatient care. Diagnostic categories included in the registry are ABI, spinal cord injury, and demyelinating diseases. The clinics are primarily focused on adults of working age, i.e., 18 and older. Data are collected as a part of the standard routine at the clinics, with the aim of securing quality of care, providing a basis for improvements, and increasing knowledge of care processes and long-term health for patient populations. Nationally, the coverage rate, i.e., the proportion of reported hospital admissions in SveReh in relation to the number of hospital admissions in the clinics, has varied since the launch in 2007 but according to SveReh it is high (29). According to the national registry centre (QRC), the registry has certification level 2 of 3, which indicates that the registry has broad coverage, supports quality improvement and research, ensures transparency and data quality, and actively incorporates the patient perspective (30). The registry contains demographic data, staff-reported rehabilitation outcome measures, results from medical assessments regarding degree of functional impairment, physical abilities, psychological health, and independence in activities of daily living (ADL), as well as patient-reported outcome measures (PROMs), and patient-reported experience measures (PREMs). In addition to outcome measures, the registry contains process measures, i.e., whether certain rehabilitation interventions have been conducted and timeliness of interventions. Data are registered on admission and discharge for both inpatients and outpatients.

### Participants

Data included in the study were for patients with ABI as main diagnosis, who had been treated in inpatient care and who had given their consent to be registered in SveReh during the period 2008 to 2020. Patients with main diagnosis other than ABI, patients who were registered as duplicated cases, i.e., cases with the same subject ID number appearing more than once, and patients whose data were insufficiently registered regarding main diagnosis and interpreter dependency were excluded.

### Procedure

National registry data from SveReh were collected from Centre of Registries Västra Götaland on 11 April 2023 (31).

### Material

*Language proficiency.* To examine interpreter dependency in relation to rehabilitation outcomes, a variable indicating language proficiency was used. The variable had 3 categories: “Swedish speaking”, “misinterpretations occur”, and “interpreter-dependent”. However, there was no information in the registry as to whether, and to what extent, an interpreter had been used for the interpreter-dependent group during their rehabilitation. The individuals categorized as “misinterpretations occur” were excluded due to unclear definition regarding their language proficiency.

*Rehabilitation outcomes*. To examine these, variables consisting of 2 validated instruments, Functional independence measure (FIM) (32) and *EQ-5D* (33), and questions from the SveReh protocol were used, presented below in relation to the aims for the study:

- *Independence in activities of daily life (ADL).* One of the aims in this study was to examine interpreter dependency in relation to independence in ADL. For this purpose, 2 variables were used:
  *Functional independence measure (FIM)*. FIM is a validated, widely accepted, 18-item-instrument to measure degree of disability during inpatient care. The instrument is summarized to a total score and is also subdivided into 2 subscales: a motor subscale (13 items covering self-care, sphincters, transfers, and locomotion) and a cognitive subscale (5 items covering communication and social cognition). The total motor subscale score ranges from 13 to 91 and the total cognitive subscale ranges from 5 to 35. Each item is scored by staff from 1 (Total assistance) to 7 (Complete independence). The maximum score on Total FIM is 126 (Full independence in all areas) and minimum score is 18 (Total assistance in all areas). Total FIM score and total subscale scores registered on admission and discharge were used in the study.
  *Discharge destination.* Second, functional independence was measured by a variable in the registry that indicated discharge destination through 9 categories, with 1 of them being “patient discharged to their own home without personal dependency”. In SveReh, personal dependency is defined as “In everyday activities being dependent on another person, such as spouse, home care service or equivalent” (34). See Table SI for full details on the discharge destination variable.
- *HRQL and self-perceived health.* Data regarding HRQL measured by the EQ-5D index and self-rated health measured by Thermometer was used. EQ-5D and Thermometer are PROMs administered as questionnaires on admission and discharge, with higher scores indicating a greater HRQL and self-rated health. The EQ-5D is validated for stroke patients (33) and areas included in the questionnaire are: mobility, self-care, usual activities, pain/discomfort, and anxiety/depression. Maximum score on EQ-5D is 1.0 (Full health) and minimum score is –0.594 (Worse than dead). Maximum score on Thermometer is 100 (The best imaginable health) and minimum score is 0 (The worst imaginable health).
- *Occurrence of complications during the period of care.* In SveReh, it is indicated whether complications have occurred during the period of care via yes or no answers. Complications included in the registry are: Covid-19 infection, deep vein thrombosis, pulmonary embolism, heterotopic bone formation, fall with fracture, contracture, epilepsy/seizures, hydrocephalus, pressure ulcers, ulcer, meningitis, pneumonia, urinary tract infection, infection with multi-resistant bacteria, other infection, new brain injury or other complication/disease/injury. If the patient absconds from the hospital ward, it is also registered as a complication.
- *Length of stay (LOS)*, measured in number of days from hospital admission to discharge.
  To examine interpreter dependency in relation to rehabilitation process measures, the following variables were used:
- *Establishment of individualized rehabilitation plan (IRP).* Swedish healthcare regions are responsible for establishing, together with the patient, an IRP for all patients in need thereof (2). In the registry, it is indicated whether the patient has received a written IRP via answers “yes”, “no” or “don’t know”.
- *Collection of PREMs.* Clinics participating in SveReh collect PREM data and register whether a patient satisfaction questionnaire has been administered or not (34), with response options “yes”, “no”, “don’t know”, and “questionnaire cannot be administered”.

### Statistical analysis

*Filtering of duplicated cases.* To avoid using the same individual more than once in the analyses, cases with the same subject ID appearing more than once were identified and filtered out before performing analyses.

### Analysis of FIM, EQ-5D, Thermometer, and LOS

- A Mann–Whitney *U* test was used to assess differences in FIM, EQ-5D, and Thermometer scores between the individuals who were interpreter-dependent and those who were not.
  These analyses were conducted using data collected on both admission and discharge. Effect size was calculated using Cohen’s *d* to analyse the degree of influence interpreter dependency had on the differences in results. An index of 0.2–0.49 indicates a small effect, 0.5–0.79 a moderate effect and *>* 0.8 a large effect, according to Cohen (35).
- The variable for LOS was calculated based on date for hospital admission and discharge. A Mann–-Whitney *U* test was then used to examine differences in LOS between the interpreter-dependent and non-interpreter-dependent group.
- A general linear mixed model was used to measure degree of improvement from admission to discharge for FIM, EQ-5D, and Thermometer scores.

*Analysis of dichotomous variables.* For variables regarding discharge destination, establishment of IRP, and collection of PREMs, new variables were created with the categories recoded to dichotomous values. Answers registered as “don’t know” were categorized as “missing data”. Together with the dichotomous variable for complications, the dichotomized outcome variables were then analysed with Pearson’s χ^2^ test.

*Controlling for confounding variables.* Logistic regression was performed for outcome variables with dichotomous values, to control for confounding variables. Demographic registry data regarding age (below/over 65 years), sex (male/female), socioeconomic background, and country of birth (Nordic/non-Nordic) were dichotomized and used as covariates. Socioeconomic background was measured by presence of income, presence of employment, and education level. No further information regarding thresholds, such as income level or extent of employment, was available. Education level was dichotomized by up to 9 years vs higher than 9 years. Age and education were dichotomized to reflect clinically meaningful groupings relevant to rehabilitation outcomes after brain injury. In particular, the cut-off at 65 years distinguishes working age from older individuals, while education level (primary vs higher) reflects differences in socioeconomic resources that may influence recovery. As a vast majority of the interpreter-dependent group were non-Nordic patients, country of birth was analysed separately from interpreter dependency, in contrast to other covariates. Odds ratio was used to measure strength of association. Total FIM scores were included in the logistic regression analysis by dichotomizing the FIM scores as over respectively under the sample median score. Imputation was applied to handle missing data.

Results with *p*-value equal to, or less than, 0.05 were considered statistically significant.

### Ethical considerations

For data collection in the national registry, patients are given written information on voluntary participation and patient confidentiality. The data are collected as part of the ongoing quality control of clinical care activities in the participating clinics. The study was approved by the Swedish Ethical Review Authority (permission number 2020-05306).

## RESULTS

**Fig. 1:**
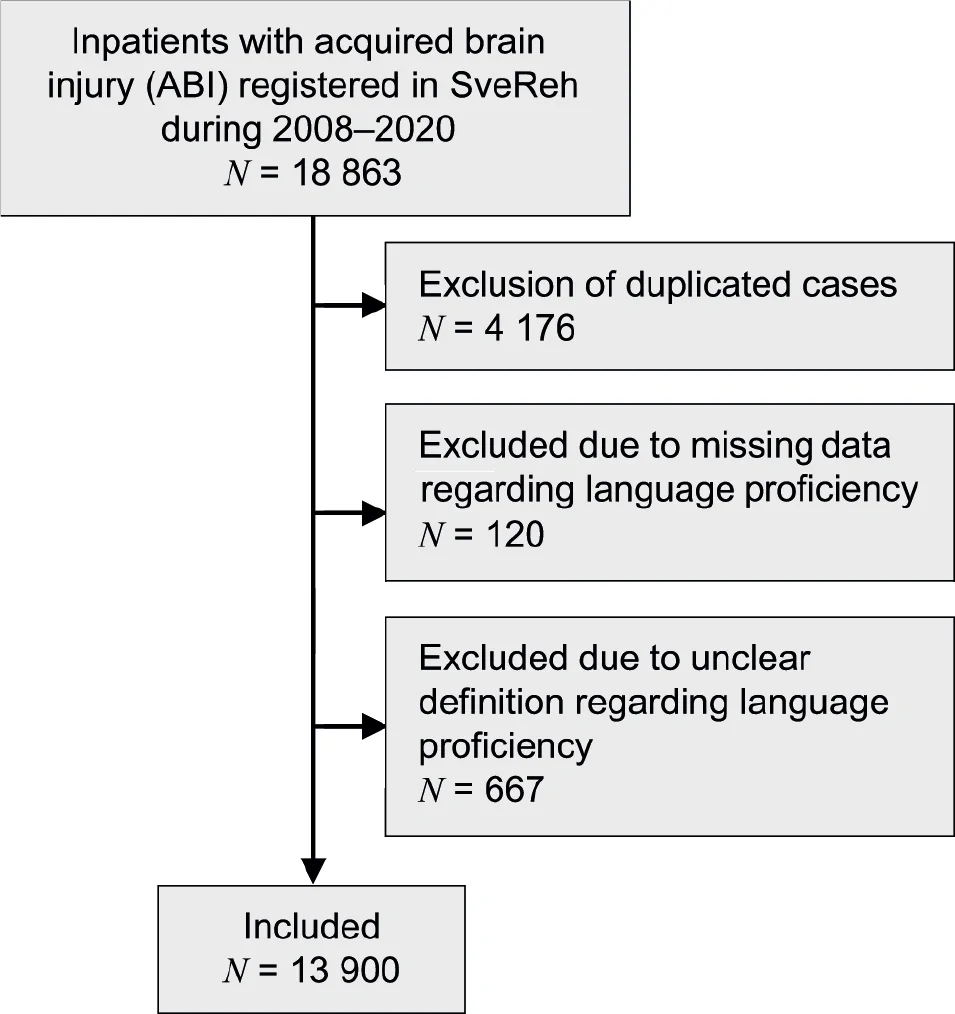
Flowchart illustrating inclusion and exclusion of data in the study, with data collected from the national quality registry SveReh.

When analysing the data set collected from the registry centre, 4,176 duplicated cases were identified, probably due to individuals being readmitted. Additionally, 120 cases were excluded due to lacking information regarding language proficiency. Furthermore, the individuals who were excluded from the study due to unclear definition regarding language proficiency amounted to 667 cases (4.6%) (Fig. 1).

Demographic and clinical data for the study participants are presented in Table I. The study population consisted of 13,900 participants, with 13,509 patients being non-interpreter-dependent and 391 being interpreter-dependent. There was a statistically significant difference in mean age between the groups, with the non-interpreter-dependent group being seven years older on average. In both groups men were overrepresented but there were no significant differences regarding sex between the groups (Table I).

**Table I:** Demographic and clinical data for study participants

Characteristics	Non-interpreter-dependent *n* = 13,509 (97.2)	Interpreter-dependent *n* = 391 (2.8)
Sex, *N* (%)		
Male	8,226 (61.0)	237 (60.6)
Female	5,583 (39.1)	154 (39.4)
Age, *N* (%)		
< 17 years	55 (0.4)	2 (0.5)
18–65 years	8,795 (65.1)	331 (84.7)*
> 66 years	4,659 (34.5)	58 (14.8)*
Age, years, mean (SD)	59.6 (15.5)	52.4 (14.2)*
Diagnosis group, *N* (%)		
Stroke (cerebrovascular disease)	8,833 (65.4)	253 (64.7)
Subarachnoid bleeding	918 (6.8)	29 (7.4)
Traumatic brain injury	1,910 (14.1)	50 (12,8)
Post infectious/post inflammatory brain injury	280 (2.1)	3 (0,^8^)
Anoxic brain injury	409 (3.0)	9 (2,3)
Other brain injury	1,159 (8.6)	47 (12,0)*
Country of birth, *N* (%)		
1. Sweden	9,598 (90.5)	8 (2.3)*
2. Other Nordic country	324 (3.1)	7 (2.0)
3. European country outside of the Nordic countries	320 (3.0)	86 (24.8)*
4. “Other country”	369 (3.5)	246 (70.9)*
5. Country of birth data missing	2,898 (21.5)	44 (11.3)*
Socioeconomic status		
1. Employment	4623 (34.6)	100 (26.1)*
2. Presence of income	12838 (96.7)	335 (91.8)*
Education < 9 years	1623 (19.4)	62 (29.2)*

* = Statistically significant difference (*p*<0.05).

### Initial statistical analysis of collected rehabilitation outcome data

*Outliers.* When analysing LOS, outliers were observed. Therefore, patients whose LOS was less than 1 day or more than 365 days were excluded before analysing LOS: 129 participants in total (126 non-interpreter dependent participants vs 3 interpreter dependent participants).

*Dropout analysis.* Total scores on EQ-5D-index and Thermometer showed a lot of missing data, ranging from 38% to 47 % depending on measure points, and a dropout analysis was conducted. It showed no statistically significant differences regarding education level (all *p* > 0.05) or gender (all *p* > 0.05). Regarding age, statistically significant differences were observed for EQ-5D and Thermometer on discharge (*p* < 0.05). However, the mean age differed by only 1 year, with the group lacking data being slightly older than the group with complete data.

For both PROMs, there were statistically significant differences on both admission and discharge regarding missingness in relation to socioeconomic variables income and employment status (*p* < 0.05), with the group lacking data having a higher proportion of individuals lacking income and employment. The proportional differences between the groups ranged from 1% to 1.9% regarding income and from 6.3% to 7.6% regarding employment. Despite the presence of statistically significant differences in socioeconomic variables between respondents and non-respondents, the dataset was considered adequate for further analyses, as the overall sample size remained large.

### Rehabilitation outcomes

When examining interpreter dependency in relation to rehabilitation outcomes, the following results were observed.

*(i) Independence in ADL:*

*FIM.* On admission, the interpreter-dependent group demonstrated a significantly lower level of independence on the total FIM, the motor subscale, and the cognitive subscale (Table II).

**Table II:** Functional independence (FIM), health-related quality of life (EQ-5D), and self-perceived health (Thermometer) in relation to interpreter dependency

	Admission				Discharge				
	Non-interpreter-dependent	Interpreter-dependent	*p-*value	Cohen’s *d*	Non-interpreter-dependent	Interpreter-dependent	*p-*value	Cohen’s *d*	Swedish population norms
Total FIM score (Min: 18 Max:126)									
*N*	12,467	360	*p* < 0.001	0.27	11,873	340	*p* < 0.001	0.28	
Mean (SD)	82.56 (32.86)	73.10 (32.79)			101.36 (27.76)	93.15 (29.66)			
Median	90	77			113	103			
Total motor subscale score (Min: 13 Max: 91)									
*N*	12,469	360	*p <* 0.001	0.19	11,876	342	*p <* 0.001	0.18	
Mean (SD)	57.81 (26.21)	52.61 (25.68)			73.08 (22.08)	68.59 (22.85)			
Median	64	55			83	77			
Total cognitive subscale score (Min:5 Max 35)									
*N*	12,468	360	*p <* 0.001	0.44	11,874	340	*p <* 0.001	0.51	
Mean (SD)	24.75 (9.11)	20.49 (9.45)			28.27 (7.47)	24.22 (8.55)			
Median	27	21			31	26			
EQ-5D index (Min: –0,594 Max: 1,0)									
*N*	8,817	202	*p <* 0.001	0.33	8,062	153	*p <* 0.001	0.53	
Mean (SD)	0.393 (0.380)	0.262 (0.395)			0.604 (0.318)	0.429 (0.409)			0.851 (0.008)
Median	0.487	0.199			0.691	0.516			
Thermometer (Min: 0 Max: 100)									
*N*	8,249	177	*p =* 0.016	0.16	7,560	134	*p <* 0.001	0.34	
Mean (SD)	53.36	49.62			65.99	58.77 (23.88)			83.3 (0.7)
Median	50	50			70	60			

The observed disparity for the total FIM and the motor subscale corresponded to only a small effect size. In contrast, the cognitive subscale showed a moderate effect size.

On discharge, the significantly lower functional independence persisted across the total FIM, motor subscale, and cognitive subscale for the interpreter-dependent group. The cognitive subscale continued to exhibit a moderate effect size, whereas the total FIM and the motor subscale showed a small effect size.

*Discharge destination.* In line with the FIM outcomes, the discharge destination results showed that the interpreter-dependent group were to a lower degree discharged to their own home without personal independence (32.9 vs 67.1), *χ*^2^ (df = 1, *N* = 13,811) = 28.8, *p <* 0.001).

*(ii) HRQL and self-perceived health:*

*EQ-5D and Thermometer.* On admission as well as discharge, the interpreter-dependent group showed significantly lower scores on EQ-5D and Thermometer (see Table II). However, effect size analysis indicated that these differences were small for both measures. On discharge, the small effect size remained for Thermometer, while EQ-5D showed a moderate effect size.

**Fig. 2:**
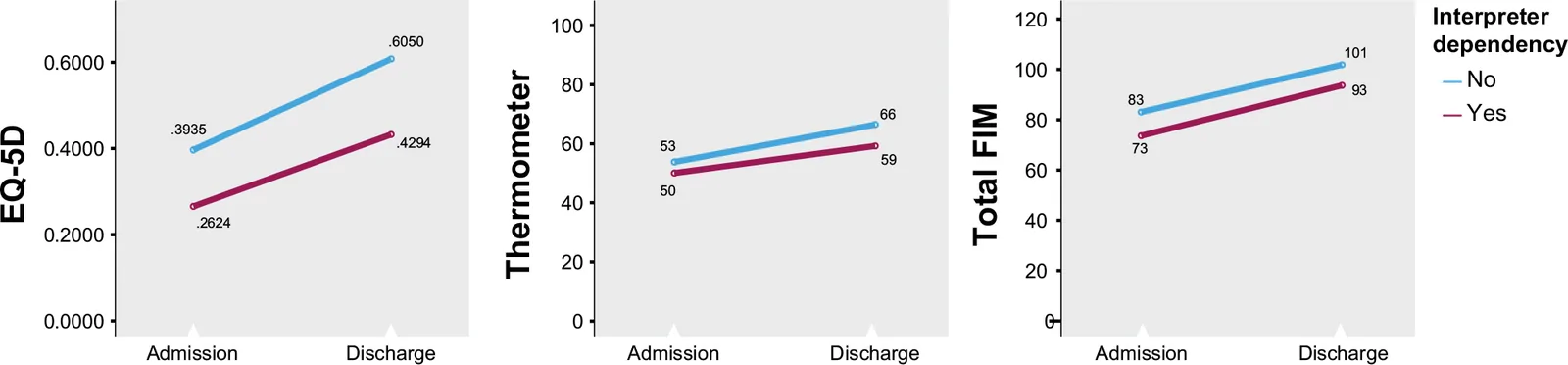
Degree of improvement from admission to discharge, for non-interpreter-dependent patients and interpreter-dependent patients. The graph illustrates rehabilitation outcomes for health-related quality of life (EQ-5D), self-perceived health (Thermometer), and functional independence (FIM).

*Degree of improvement.* Degree of improvement from admission to discharge was assessed for total FIM, EQ‑5D, and Thermometer. Admission/discharge counts were 12,467/11,873 vs 360/340 (FIM), 8,817/8,062 vs 202/153 (EQ‑5D), and 8,249/7,560 vs 177/134 (Thermometer) for non‑-interpreter dependent and interpreter‑dependent groups, respectively. Regardless of interpreter dependency, the patients improved their functional independence (*p <* 0.001), HRQL (*p* < 0.001) and self-perceived health (*p* < 0.001) after going through rehabilitation. There was no statistically significant relationship between interpreter dependency and degree of improvement of functional independence (*p* = 0.6), HRQL (*p* = 0.3) and self-perceived health (*p* = 0.2) (Fig. 2).

*(iii) Occurrence of complications:*

There was no significant difference regarding complications χ^2^ (df = 1, N = 13,900) = 1.62, *p =* 0.206.

*(iv) Length of stay (LOS):*

No significant relationship between interpreter dependency and LOS was observed (*p =* 0.96)*.*

### Rehabilitation process measures

When exploring interpreter dependency in relation to rehabilitation process measures, the following results were found.

*(v) Establishment of IRP:*

There was no significant difference between the 2 groups regarding establishment of IRP, χ^2^ (df = 1, *N* = 13,735) = 0.31, *p* = 0.58).

*(vi) Collection of PREMs*:

For the interpreter-dependent group, the staff had to a lesser extent registered “yes” to the question as to whether PREMs had been completed, χ^2^ (df = 1, *N* = 11,699) = 93.44, *p <* 0.001). In the same way, the option “questionnaire cannot be administered” was reported to a greater extent for the interpreter-dependent group, χ^2^ (df = 1, *N* = 11,699) = 116.41, *p* < 0.001).

### Controlling for confounding variables

As presented in Table III, the observed relationship between interpreter dependency and rehabilitation outcomes and process measures persisted after adjusting for age, sex, income, employment, and education level.

**Table III:** Interpreter dependency and country of birth in relation to rehabilitation outcome and process measures, adjusting for sex, age, income, employment, and education level

Factor	Interpreter-dependent, adjusted OR (95% CI), p-value	Non-Nordic country of birth, adjusted OR (95% CI), p-value
Rehabilitation outcome measures		
Independence in everyday life		
Total FIM score over median on admission	OR: 0.59 (CI: 0.48–0.73), *p <* 0.001	OR: 0.99 (CI: 0.90–1.09), *p =* 0.849
Total FIM score over median on discharge	OR: 0.45 (CI: 0.36–0.56), *p <* 0.001	OR: 0.80 (CI: 0.72–0.88), *p <* 0.001
Discharge destination registered as “independent and discharged to their own home”	OR: 0.58 (0.47–0.72), *p <* 0.001	OR: 0.80 (0.72–0.89), *p <* 0.001
Rehabilitation process measures		
PREMs collected registered as “yes”	OR: 0.40 (CI: 0.33–0.49), *p <* 0.001	OR: 0.69 (CI: 0.62–0.76), *p <* 0.001
PREMs registered as “questionnaire cannot be administered”	OR: 3.73 (CI: 2.84–4.84), *p <* 0.001	OR: 1.92 (CI: 1.61–2.27), *p <* 0.001

OR: odds ratio; CI: confidence interval.

While interpreter dependency was linked to lower independence on admission, no such association was found for individuals born outside the Nordic region. However, as with interpreter dependency, being born outside the Nordic region was associated with lower independence on discharge and a reduced likelihood of having completed PREMs (see Table III).

## DISCUSSION

This registry-based study aimed to increase knowledge regarding neurorehabilitation outcomes and process measures in relation to interpreter dependency. Analysis of admission and discharge data showed that interpreter dependency was related to lower scores regarding functional independence, HRQL, and self-perceived health. However, there was no association between interpreter dependency and the degree of improvement in these outcomes between the 2 time points. Discharge destination analysis showed that the interpreter-dependent group were to a higher degree discharged to their own home, being dependent on support from family or community services. Furthermore, PREMs were administered to a lower degree with interpreter-dependent patients. The observed disparities persisted after adjusting for age, sex, and socioeconomic variables.

### Rehabilitation outcomes

*Degree of improvement in functionality and HRQL.* Despite challenges reported in previous research, this study showed no relationship between interpreter dependency and degree of improvement regarding functional independence, HRQL, and self-perceived health. However, similar to the results observed by Mellahn et al. (16), interpreter-dependent patients presented lower functional independence and HRQL already on admission. These results are noteworthy and should be further explored, as the results could be connected to risk factors or disparities for interpreter-dependent patients earlier in the care continuum, especially considering previous research showing that CALD patients face longer LOS in the emergency department (4), delayed interventions (36), lower health literacy (14), a higher proportion of severe ABI (37), and increased risk of disability (38). The disparities observed on admission in the present study indicate that interpreter-dependent patients may be in greater need of thorough preventive care and that measures should be implemented to ensure equitable communication throughout the entire continuum of care. This could include healthcare staff training in interpreter-mediated communication and culturally competent care, improving access to professional interpreters, and translation of written information. Improving the work environment in healthcare, in terms of adequate staffing, collegial support, and managerial support, has also been found to improve outcomes for patients with low proficiency in the majority language (39). Other implementations could include adapting rehabilitation to be provided in more culturally familiar formats and increased focus on including the family in the rehabilitation process.

*Functional independence in daily living.* In line with the limited existing research (16, 17), interpreter dependency was related to lower functional independence after ABI. This relation was observed on admission as well as on discharge. Contrary to previous research focusing on low proficiency in the majority language (40), but in line with Mellahn et al. (16), the relationship was observed also regarding the motor FIM scores. However, the improvement and effect size for differences in motor function was small. Results regarding discharge destination, showing that the interpreter-dependent group was to a lower degree discharged to their own home without personal dependency, supported previous research (16, 36). These findings suggest that interpreter-dependent patients could benefit from extended support and targeted interventions within outpatient rehabilitation.

When studying the concept “independence” as an outcome after ABI, cultural norms should be considered. Views on the role of family members can vary across cultures, with varying degrees of promotion of independence (41). However, in the present study, interpreter dependency was more strongly associated with lower FIM scores than was country of birth. This indicates that language barriers may play an important part and that differences in outcome should not be attributed solely to cultural aspects.

*HRQL and self-perceived health.* As the conducted dropout analysis for EQ-5D and Thermometer showed a statistically significant difference between the groups regarding socioeconomic variables, there is a risk that the patients with registered data were not representative enough for the whole group. Additionally, the effect sizes of observed differences were small, indicating limited practical significance. As the interpreter-dependent group reported lower scores on PROMs (EQ-5D and Thermometer) on admission as well as on discharge, conducting PROMs with interpreter-dependent patients could be worth exploring further in clinical evaluation and rehabilitation research. Standardized tests are important tools for assessing functional impairments after ABI, but it is equally important to collect information concerning the patient’s own experience of their health and function. PROMs have the potential to capture aspects of health and functioning that may otherwise be overlooked with interpreter-dependent patients, thereby contributing to more person-centred and equitable care. A structured approach with additional time allocated for interpreter-dependent patients may facilitate PROM collection and improve response rate. The use of visual aids could further support patient understanding of questionnaire items, helping overcome language barriers and facilitate data collection and quality.

*Complications.* In line with Mellahn et al. (17), interpreter dependency was not related to a higher occurrence of complications. Type-specific complication data could have added clearer information, but these data were not available in our dataset. Although these are encouraging findings, it stands in contrast to previous research that has identified a higher degree of complications and safety risks linked to language barriers (26, 42). Possible reasons for these variations may include demographic differences, healthcare organizational factors, rehabilitation phase, staff-to-patient-ratio, participant age, and the patients’ socio-legal status. Tax-funded healthcare systems, such as the Swedish model, are guided by principles of equal access and need-based care, which may decrease the risk of disparities regarding complications. This warrants further exploration. As stressed in previous literature, well-functioning communication in healthcare is central for patient safety (43).

*LOS.* The LOS result, showing no relationship between interpreter dependency and LOS, is in contrast to previous studies focusing on language barriers (14, 16) and in line with a study focusing on CALD patients (18). When analysing LOS, the lack of information in the registry on interpreter use was suboptimal. The LOS result is noteworthy, considering that interpreted communication by nature is time-consuming. Previous studies have shown that patients interacting with professional interpreters had longer LOS together with larger rehabilitation gains compared with patients supported by ad-hoc interpreters (13). This result may indicate that professional interpreters were not used to a sufficient extent. It is also possible that the clinics participating in the SveReh registry need to follow care protocols with standard LOS, making it challenging to offer patient-centred care for interpreter-dependent patients.

### Rehabilitation process measures

*Collection of PREMs.* PREMs were collected to a markedly lesser extent with the interpreter-dependent group. The fact that the option “questionnaire cannot be administered” was registered to a significantly higher degree for the interpreter-dependent group indicates difficulties in conducting the survey. These results might mirror the lack of access and accessibility to interpreters (7), making PREM data collection time-consuming and deprioritized for interpreter-dependent patients. However, considering the fact that CALD patients constitute an increasingly higher number of the Swedish patient population, it should be in the interest of rehabilitation clinics to include them in PREM data collection. From a patient perspective, collection of PREMs should not be restricted due to interpreter need, if aiming for equal care.

Regarding process measures, this study is limited by the inclusion of only 2 process measures. A more comprehensive evaluation of rehabilitation processes would require the incorporation of additional measures, for example data showing whether the patients have been assessed by specific healthcare professionals. However, these data were not available.

### Confounding variables

A challenge in epidemiology is the complex interaction between clinical and demographic characteristics. Efforts were made to adjust for socioeconomic variables. However, considering the major ethnic disparities in Sweden regarding socioeconomic status, with poverty being 7 times more common for people born outside of Sweden (44), it is likely that the socioeconomic variables used in the study were insufficient for identifying confounding. Additionally, factors such as age and degree of impairment are known to influence the level of independence after rehabilitation. It would therefore have been optimal to include data on degree of ABI impairment as an additional covariate in the logistic regression analysis, but it was not possible to fully assess that with our dataset. It is thus possible that difficulties related to ABI severity could explain observed differences in rehabilitation outcomes. Mellahn et al. identified a higher proportion of haemorrhagic strokes and lower independence on discharge among interpreter-dependent patients (17). However, they concluded that when adjusting for stroke type, interpreter-dependent patients continued to exhibit significantly lower rates of independence.

### Methodological considerations

When interpreting the results of this study, some limitations must be considered. First, this is a real-life study conducted in a clinical setting, which entails both strengths and limitations. The study relied on data collected and registered by clinical and administrative staff and the purpose of collecting the data in national quality registries is primarily to measure healthcare quality. Second, registry-based studies, such as the present study, include large sample sizes, with the risk that small differences may lead to significant results with limited practical significance. Third, missing data for the variables related to HRQL and country of birth could potentially affect the results regarding rehabilitation outcomes and the control of confounding variables.

*Participants.* As mentioned, the coverage rate for the registry is stated to be high according to SveReh (29). A limitation in this study is the lack of more detailed information regarding national coverage rate across the entire study period, which may be associated with a risk that the study population does not represent the true population. Regarding language proficiency, the variable was roughly divided in the registry into 3 categories, with unclear definitions. This introduces a risk that participants who were excluded due to being classified in the category “misinterpretations occur” may in fact have been interpreter-dependent and therefore potentially should have been included in the study. As previous research has shown, there is a risk that patients rely on “getting by” without an interpreter despite difficulties communicating in the majority language (45). Regarding language background, the variable was restricted to 4 predefined categories, which limited the ability to explore differences between specific language groups. The fact that there was no information in the registry on whether, and to what extent, an interpreter had been used for the interpreter-dependent group reduces our level of confidence in the robustness of our conclusions. Additionally, among the staff who met the interpreter-dependent patients in the present study, the experience and proficiency in interpreter-mediated communication have likely varied. This challenge is described in previous research that has identified a lack of information on interpreter usage and availability (14). Previous studies have recommended more detailed documentation of interpreter service usage to gain insight into medical outcomes for the interpreter-dependent patients (10).

*Cultural and linguistic adaptation of instruments.* There is a risk that PROM and PREM instruments are not properly adapted linguistically or culturally for the interpreter-dependent group. When using PROMs and PREMs, there is also a risk of selection bias. As Calvert et al. point out, exclusion of ethnic minority groups from PREMs, due to lack of linguistically validated and culturally appropriate instruments, threatens to increase ethnic disparities (46). In conclusion, this study shows that interpreter-dependent patients may benefit from rehabilitation to a similar degree to non-interpreter-dependent patients. However, interpreter-dependent patients may be a vulnerable patient group in neurorehabilitation, as they showed lower functional independence and lower HRQL on admission as well as discharge. Possible healthcare disparities earlier in the care continuum should be further explored. Additionally, PREMs were conducted to a lesser extent among interpreter-dependent patients, limiting the equity and representativeness of patient experience data. These findings support previous studies that show healthcare disparities in relation to low proficiency in the majority language. We suggest that clinics and quality registries document detailed data regarding language proficiency, interpreter use, and ABI severity, in order to strive for equitable care and enable further research in this area.

## Supplementary Material


