# Supplementary material for: ASSOCIATIONS BETWEEN INTERPRETER DEPENDENCY AND REHABILITATION OUTCOMES AND CARE PROCESSES FOLLOWING ACQUIRED BRAIN INJURY: A SWEDISH REGISTRY-BASED COHORT STUDY
Source: J Rehabil Med. 2026 Sep 14;58:45146. doi: 10.2340/jrm.v58.45146 (PMC13583992; doi:10.2340/jrm.v58.45146)
Supplement: Supplementary file 1 [file JRM-58-45146-s1.pdf]

Supplementary table I. Discharge destination variables in the SveReh registry

	**Non-interpreter dependent**<br>N (%)<br>13,509 (97.2)	**Interpreter dependent**<br>N (%)<br>391 (2.8)
Discharge destination N (%)		
Own home without personal independence	6,264 (46.7)	128 (32.9) *
Own home with personal independence	4,417 (32.9)	162 (41.6) *
Specialized residential care facility	1,716 (12.8)	58 (14.9)
Other rehabilitation unit	413 (3.1)	10 (2.6)
Discontinued rehabilitation due to own decision	61 (0.5)	4 (1.0)
Discontinued rehabilitation due to medical reasons	268 (2.0)	11 (2.8)
Deceased	43 (0.3)	0 (0.0)
Other	240 (1.8)	16 (4.1) *

* = Statistically significant difference ($p < 0.05$)
